# Cardiopulmonary resuscitation traumatic cardiac arrest - there are survivors. An analysis of two national emergency registries

**DOI:** 10.1186/cc10558

**Published:** 2011-11-22

**Authors:** Jan-Thorsten Gräsner, Jan Wnent, Stephan Seewald, Patrick Meybohm, Matthias Fischer, Thomas Paffrath, Arasch Wafaisade, Berthold Bein, Rolf Lefering

**Affiliations:** 1 Department of Anaesthesiology and Intensive Care Medicine, University Hospital Schleswig-Holstein, Campus Kiel, Schwanenweg 21, Kiel, 24105, Germany; 2 Department of Anaesthesiology and Intensive Care Medicine, University Hospital Schleswig-Holstein, Campus Lübeck, Ratzeburger Allee 160, Lübeck, 23538, Germany; 3 Department of Anaesthesiology and Intensive Care, Klinikum am Eichert,Eichertstraße 3, Göppingen, 73035, Germany; 4 Department of Orthopedic and Trauma Surgery, Cologne Merheim Medical Center, Ostmerheimerstr. 200, Cologne, 51109, Germany; 5 Institute for Research in Operative Medicine, Faculty of Health, University of Witten/Herdecke, Alfred-Herrhausen-Straße 50, Witten, 58448, Germany

## Abstract

**Introduction:**

Cardiac arrest following trauma occurs infrequently compared with cardiac aetiology. Within the German Resuscitation Registry a traumatic cause is documented in about 3% of cardiac arrest patients. Regarding the national Trauma Registry, only a few of these trauma patients with cardiac arrest survive. The aim of the present study was to analyze the outcome of cardiopulmonary resuscitation (CPR) after traumatic cardiac arrest by combining data from two different large national registries in Germany.

**Methods:**

This study includes 368 trauma patients (2.8%) out of 13,329 cardiac arrest patients registered within the Resuscitation Registry, whereby 3,673 patients with a cardiac cause and successful CPR served as a cardiac control group. We further analyzed a second group of 1,535 trauma patients with cardiac arrest and early CPR registered within the Trauma Registry, whereby a total of 25,366 trauma patients without any CPR attempts served as a trauma control group. The relative frequencies from each database were used to calculate relative percentages for patients with traumatic cardiac arrest in whom resuscitation was attempted.

**Results:**

Within the Resuscitation Registry, cardiac arrest was present in 331 patients (89.9%) when the EMS personal arrived at the scene and in 37 patients (10.1%) when cardiac arrest occurred after arrival. Spontaneous circulation could be achieved in 107 patients (29.1%). A total of 101 (27.4%) were transferred to hospital, 95 of whom (25.8%) had return of spontaneous circulation (ROSC) on admission. According to the Trauma Registry, the overall hospital mortality rate for cardiac arrest patients following trauma was 73% (*n *= 593 of 814). About half of the patients who were admitted alive to hospital died within 24 hours, resulting in 13% survivors within 24 hours. 7% of the patients survived until hospital discharge, and only 2% of the patients had good neurological outcome.

**Conclusions:**

Our present study encourages CPR attempts in cardiac arrest patients following severe trauma. When a manageable number of patients is present, the decision on whether to start CPR or not should be done liberally, using comparable criteria as in patients with cardiac etiology. In this respect, trauma management programs that restrict CPR attempts should not be encouraged.

## Introduction

Cardiac arrest following trauma occurs relatively rarely in comparison with cardiac or other etiologies. Within the German Resuscitation Registry (GRR) that is managed by the German Society of Anaesthesiology and Intensive Care Medicine (*Deutsche Gesellschaft für Anästhesiologie und Intensivmedizin*, DGAI), traumatic cause is documented in about 3% of cardiac arrest patients [1].

Pre-hospital cardiopulmonary resuscitation (CPR) is performed infrequently in patients with severe trauma. The Trauma Registry of the German Society for Trauma Surgery (*Deutsche Gesellschaft für Unfallchirurgie*, TR-DGU) registers patients who had severe injuries with a potential need for intensive care and who had spontaneous circulation on admission. Approximately 3% of these severely injured patients documented within the TR-DGU received CPR attempts outside the hospital. Only a few of these patients survived [2], and only 1 out of 10 patients with pre-hospital CPR attempts achieved a good outcome [3].

There is an ongoing debate in terms of the effectiveness of CPR in trauma patients, particularly with regard to good long-term outcomes [4-6]. International trauma training courses have even suggested that no intervention should be started in cardiac arrest patients with primary asystole due to traumatic causes [3].

Several factors are known to influence the success of CPR. The most important factor is time. If cardiac arrest occurs during pre-hospital treatment and is observed by an emergency physician, intervention and transport should be started without any delay. Other pre-hospital factors influencing the primary goal of CPR - the return of spontaneous circulation (ROSC) - have recently been analyzed and identified within the GRR [7]. General prognostic factors known to influence survival after trauma, such as age, blood loss, and the severity of injury, also affect this subgroup of trauma patients.

Since the TR-DGU, however, is limited to trauma patients who had spontaneous circulation at hospital admission, we now combine data from two different large national registries mentioned above - the GRR and the TR-DGU - to analyze the success rate of CPR after traumatic cardiac arrest in Germany.

Design and publication of this study were approved by the scientific committee of the GRR and the TR-DGU in compliance with current publication guidelines. This study was approved by the ethics committee of the University of Cologne, Faculty of Medicine (Kerpener Str. 62, 50937 Cologne, Germany) (Register Number 11-014) and the ethics committee of the University of Kiel, Faculty of Medicine (Schwanenweg 20,24105 Kiel, Germany) (Register Number D456/11).

## Materials and methods

### German Resuscitation Registry (GRR)

The GRR currently represents 51 emergency medical systems that record data on out-of-hospital CPR attempts throughout the country, covering a population of nine million citizens (the total population of Germany is 85 million). Participation is voluntary. In Germany, emergency medical systems (EMS) are staffed by emergency physicians from several medical specialties (mainly anesthesiology, surgery, and internal medicine) who had additional training in emergency medicine. The registry is organized and funded by the DGAI [8].

The GRR is divided into two different data sets. Firstly, a 'preclinical care' data set derived from the Utstein-style template for uniform reporting of cardiac arrest, aiming at documentation of pre-hospital logistic issues, presumed aetiology, resuscitation therapy and the patient's initial outcome, including 118 variables. Secondly, the 'post-resuscitation care' data set is aimed at documenting in-hospital post-resuscitation efforts. Due to the anonymity of data collection and the fact that the primary purpose of the GRR is quality control, patient consent was not necessary [1]. ROSC was defined as a palpable pulse for more than 20 seconds [9,10]. Admission to hospital (ATH) was regarded as a positive outcome if circulation was still present on hospital admission (group A_GRR_). Failure of pre-hospital ROSC or ongoing CPR on admission was defined as a negative outcome (no ROSC/no ATH; group B).

Within the GRR 13,329 out-of-hospital cardiac arrest patients were prospectively documented between 1998 and 2010 for which a professional pre-hospital EMS team was requested by dispatchers. The present study includes 368 patients (2.8%) with cardiac arrest most probably due to traumatic cause; 3673 cardiac arrest patients with a cardiac cause and with ROSC at hospital admission served as a 'cardiac control group' (group C).

Patients from the GRR were divided into the following three groups:

• group A_GRR_: pre-hospital CPR with ATH (*n *= 95)

• group B: pre-hospital CPR without ROSC/ATH (*n *= 273)

• group C: cardiac control group with ROSC (*n *= 3,673).

### Trauma Registry of the German Society for Trauma Surgery (TR-DGU)

The TR-DGU is a prospective structured database established in 1993. Participation has been voluntary until recently, when it became an obligatory tool for quality assessment in regional trauma networks [11]. Data from the pre-hospital phase, early in-hospital phase (emergency department), ICU and hospital care until discharge from hospital are collected in an anonymous fashion. Patients who had severe injuries with a potential need for intensive care and who had spontaneous circulation on admission, were included. In contrast to the GRR, patients with burns, drowning, poisoning and preclinical deaths were excluded. Data were documented within the TR-DGU via a password-protected online program with multiple plausibility and completeness checks. Participating hospitals received extended annual audit reports in which results were compared across institutions. More than 200 hospitals are currently participating in this registry, providing about 10,000 patients per year. The Trauma Registry is organized by the DGU. Due to the anonymity of data collection and the fact that the primary purpose of the registry is quality control, patient consent was not necessary.

For the present analysis, 1,535 patients with early CPR were included. The inclusion criteria were as follows: primarily admission from the pre-hospital site of injury; Injury Severity Score (ISS) of nine points or more; admission to a hospital in Germany; available data about pre-hospital and early in-hospital CPR attempts (performed/not performed); year of injury from 1993 to 2009. Of included patients, 814 received pre-hospital CPR, while 989 patients experienced cardiac arrest during the early in-hospital phase before ICU admission, and 268 patients had both pre-hospital and in-hospital CPR attempts. A total of 25,366 patients from the TR-DGU with the same inclusion criteria, but without any CPR attempts, served as a 'trauma control group' (group E).

Patients from the TR-DGU were divided into the following three groups:

• group A_TR-DGU_: pre-hospital CPR and ATH (*n *= 814)

• group D: trauma control group without any CPR (*n *= 25,366)

Group A_TR-DGU _from the TR-DGU corresponds to group A_GRR _from the GRR, which lists patients who were admitted to a hospital with circulation after cardiac arrest.

The GRR represents all patients with any pre-hospital cardiac arrest at the participating centers who received CPR treatment by emergency medical doctors, independently of ROSC. TR-DGU represents only patients with severe trauma who reached hospital with spontaneous circulation. Due to data security and confidentiality, there is no information available about whether or not individual patients were included in both registries in parallel. As the GRR does not provide any information about the in-hospital treatment and outcome after cardiac arrest and the TR-DGU is limited to trauma patients who had spontaneous circulation at hospital admission but does not contain data of patients who died at the scene, we combined data from these two large national registries in this study to analyze the mid-term outcome of CPR after traumatic cardiac arrest in Germany.

### Statistics

Data are presented as means ± standard deviation (SD) for continuous variables and as percentages for categorical variables. Due to the multiplicity of groups and variables, together with the large sample size, statistical tests were limited to a few specific conditions. Continuous variables were compared using the non-parametric *U *test, while frequencies were compared using the chi-squared test. In the final combination of results, the relative frequencies from each data source were used to calculate relative percentages for patients with traumatic cardiac arrest in whom CPR was attempted (100%). A *P *value less than 0.05 was regarded as statistically significant. Two-tailed tests were applied. All of the analyses were performed with SPSS, version 18 (IBM Corporation, Somers, NY, USA).

## Results and discussion

### GRR

**Figure 1:**
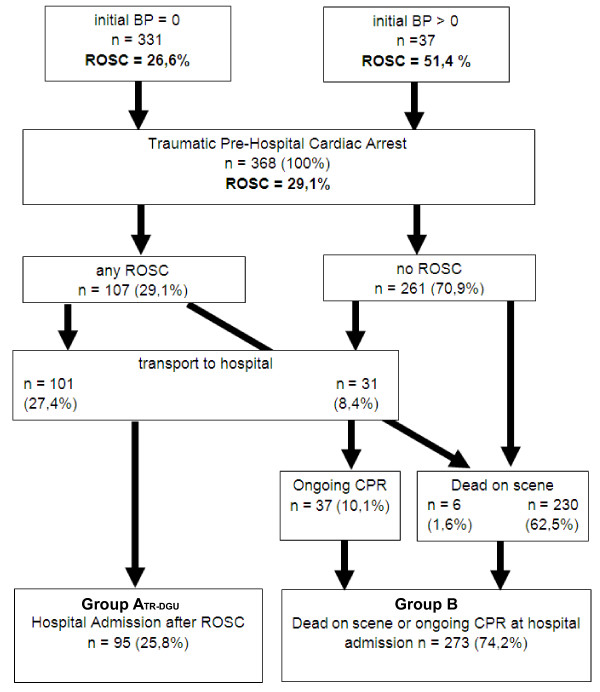
**Results for 368 patients with traumatic cardiac arrest and pre-hospital CPR based on the German Resuscitation Registry (GRR)**. BP, blood pressure; CPR, cardiopulmonary resuscitation; ROSC, return of spontaneous circulation.

Of 13,329 documented patients within the GRR database, 368 (2.8%) patients were identified with cardiac arrest due to trauma. In 331 of these patients (89.9%), cardiac arrest was present when the EMS personnel arrived at the scene, and in 37 patients (10.1%), cardiac arrest occurred after arrival at the scene during medical treatment. ROSC could be achieved in 107 patients (29.1%). A total of 101 patients (27.4%) were transferred to a hospital, 95 of whom (25.8%) had circulation on admission (group A_GRR_; Table 1 and Figure 1), while six patients died during transfer. No ROSC at any time was observed in 261 patients (70.9%). Patients who died at the scene or during transfer, and those who were admitted with ongoing CPR, were assigned to group B (*n *= 273, 74.2%; Figure 1 and Table 1). Thus, approximately one of four patients with traumatic cardiac arrest in whom CPR was initiated reached hospital with spontaneous circulation.

**Table 1:** Patient characteristics, circumstances, and treatment of patients with cardiac arrest after trauma based on the German Resuscitation Registry

	**A**_**GRR **_**Hospital admission with ROSC**	B Died at the scene or with ongoing CPR	C Hospital admission; cardiac causes
Patients (n)	95	273	3673
Age (mean ± SD)	52.7 ± 22.8	50.7 ± 22.2	67.0 ± 15.1
Aged over 60 years	44.9%	38.1%	72.4%
Age cohorts			
1-20 years	12.4%	10.0%	1.3%
21-40 years	16.9%	24.5%	3.4%
41-60 years	25.8%	29.0%	24.4%
61-80 years	36.0%	29.0%	54.2%
> 80 years	9.0%	7.4%	16.6%
Male gender	66.0%	74.8%	68.8%
Scene of cardiac arrest			
Home	27.4%	21.0%	58.6%
Nursing home	2.1%	2.2%	2.9%
Workplace	3.2%	4.9%	2.6%
Street	32.6%	46.1%	9.9%
Public place	16.8%	15.0%	12.7%
Medical institution	1.1%	1.9%	5.1%
Public event	1.5%	0.6%	
Other	14.7%	7.5%	7.7%
ECG findings			
Ventricular fibrillation	16.3%	4.4%	52.0%
PEA	13.8%	20.6%	10.0%
Asystole	57.5%	66.7%	28.4%
Other	12.6%	8.4%	9.6%
Cardiac arrest witnessed			
No	33.7%	45.1%	25.4%
By lay people	50.5%	46.9%	59.3%
By EMS	15.8%	8.1%	15.3%
Bystander CPR	16.0%	13.2%	21.6%
Use of defibrillator	31.6%	26.4%	70.1%
Fluid resuscitation (mL; mean ± SD)			
Total volume	1194 ± 1020	890 ± 1092	577 ± -423
Crystalloids	837 ± 712	585 ± 628	549 ± 395
Colloids	319 ± 486	300 ± 562	25.8 ± 117
Hyperoncotic solutions	46.9 ± 111	48.6 ± 107	11.4 ± 50.2
Time intervals (min; mean ± SD)			
Time from call to EMS arrival	9.0 ± 6.4	8.3 ± 5.0	7.8 ± 5.3
Time from accident to hospital admission	55.1 ± 15.5	65.1 ± 68.0	56.7 ± 21.9

Group A_GRR _had ROSC at hospital admission, while group B did not have any ROSC or died at the scene. The cardiac control group, group C, had cardiac causes for cardiac arrest and reached hospital with ROSC. CPR, cardiopulmonary resuscitation; ECG, electrocardiography; EMS, emergency medical service; PEA, pulseless electrical activity; ROSC, return of spontaneous circulation; SD, standard deviation.

In comparison with the group of patients in whom ROSC could not be achieved (group B), differences were observed with regard to the electrocardiography (ECG) findings (there were more patients with ventricular fibrillation who survived) and whether or not the event was observed (there were more survivors if it was observed).

Trauma patients who reached hospital with circulation after ROSC differed from the corresponding cardiac control patients who had ROSC (group C) in several aspects: trauma patients were younger, asystole was the predominant ECG finding (*P *< 0.001, chi-squared test), and cardiac arrest occurred more frequently at a public place (e.g. workplace, street, public areas: 52.6% versus 25.2%) than at home (27.4% versus 58.6%; *P *< 0.001, chi-squared test). Patients with traumatic cardiac arrest had a mean age of 52.7 years (± 22.8 years) in comparison with 67.8 years (± 15.1 years) if there was a cardiac cause (*P *< 0.001, *U *test). Bystander CPR was only performed in 16% of trauma patients compared with 21.6% of cardiac arrest patients with cardiac causes (*P *= 0.273, chi-squared test).

### TR-DGU

**Figure 2:**
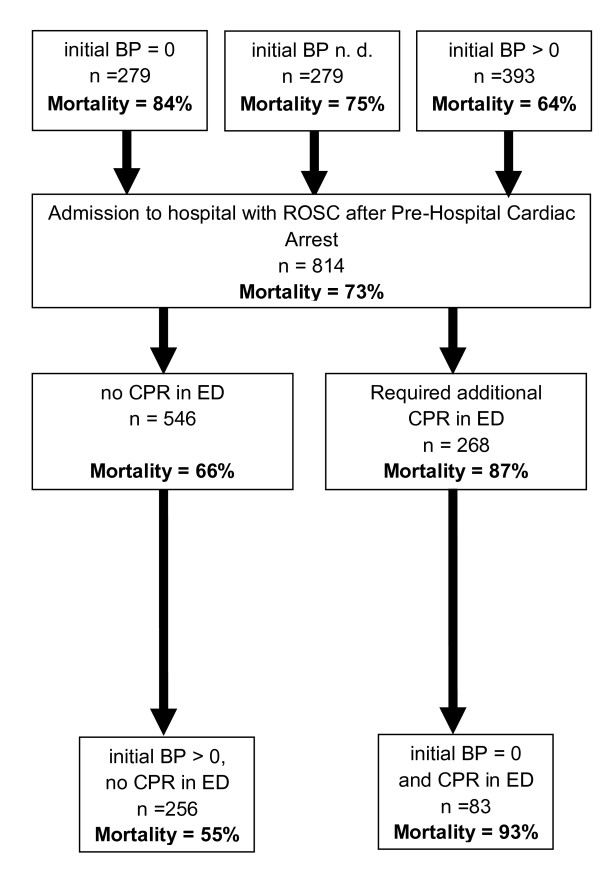
**Overview of hospital mortality rates, based on cardiopulmonary resuscitation (pre-hospital) and initial circulation (blood pressure)**. BP, blood pressure; CPR, cardiopulmonary resuscitation; ED, emergency department; n.d., not documented; ROSC, return of spontaneous circulation.

The overall hospital mortality rate for trauma cardiac arrest patients was 73% (*n *= 593 of 814; Table 2 group A_TR-DGU_). Patients who were found without any circulation at the initial pre-hospital assessment had an even poorer outcome (*n *= 279; mortality rate 84%), whereas the initial presence of blood pressure was more beneficial (*n *= 279; mortality rate 64%; Figure 2).

**Table 2:** Characteristics, treatment, and outcome of patients with severe trauma and cardiac arrest based on the Trauma Registry

	**A**_**TR-DGU **_**Pre-hospital resuscitation**	DNo resuscitation
Patients (n)	814	25,366
Age	44.1 ± 21.8	42.2 ± 20.6
Aged > 60 years	28.3%	22.4%
Male gender	72.2%	72.9%
Injury Severity Score	39.9 ± 19.7	24.0 ± 12.5
Head injury (AIS ≥3)	74.6%	47.5%
Penetrating trauma	6.4%	4.7%
Suicide (suspected)	7.6%	5.8%
Mechanism of injury		
Traffic	60.9%	62.9%
High fall (> 3 m)	13.4%	16.7%
Initial blood pressure (mmHg)	56 ± 56	121 ± 31
Shock (BP ≤ 90 mmHg)	72.3%	17.3%
Cardiac arrest on arrival (BP = 0 mmHg)	41.5%	0%
Heart rate (beats/min)	65 ± 55	95 ± 21
Endotracheal intubation	97.7%	53.8%
Chest drain	17.1%	5.9%
Catecholamines	77.5%	5.2%
Volume administration	93.7%	92.3%
Volume (total, mL)	1886 ± 1382	1372 ± 1056
Crystalloids (mL) *	1306 ± 901	1061 ± 697
Colloids (mL) *	971 ± 688	786 ± 516
Hyperoncotic solutions (mL) *	347 ± 222	328 ± 238
Time at scene (arrival to departure)	34.6 ± 17.1	32.6 ± 19.2
Time from accident to hospital admission	66.2 ± 30.8	69.7 ± 36.4
Transportation by helicopter	38.1%	40.1%
Blood pressure (mmHg)	96 ± 43	125 ± 29
Shock (BP ≤ 90 mmHg)	44.7%	11.6%
Heart rate (beats/min)	98 ± 34	90 ± 20
Blood transfusion	44.7%	27.6%
Mass transfusion	15.4%	7.1%
Cardiopulmonary resuscitation	36.1%	0%
Emergency surgery	14.5%	6.3%
Any surgery	57.7%	80.5%
24-hour mortality	51.4%	5.5%
Hospital mortality	72.9%	12.5%
Length of hospital stay in survivors (days; mean ± SD)	33.4 ± 30.6	29.1 ± 27.8
Length of hospital stay in non-survivors (days; mean ± SD)	3.9 ± 8.9	8.4 ± 15.4
GOS **		
Good recovery	8.8%	46.2%
Minor limitations	6.5%	26.6%
Severe limitations	5.9%	11.7%
PVS	4.9%	2.4%
Discharged at home	6.9%	38.6%
Discharged at home among survivors	24.3%	44.1%

The trauma control group had no cardiac arrest until ICU admission.

AIS, Abbreviated Injury Scale; BP, blood pressure; GOS, Glasgow Outcome Scale; PVS, persistent vegetative state; SD, standard deviation. * If this type of volume was administered. ** The GOS has only been available since 2002, and percentages therefore do not add up to exactly 100% with hospital mortality.

Approximately one of three patients (*n *= 268; 33%) required additional CPR during initial treatment after hospital admission. These patients had a poorer outcome (mortality rate 87%) than those who did not require any additional in-hospital CPR attempts (mortality rate 66%; Figure 2).

Patients who received pre-hospital and in-hospital CPR and in whom blood pressure was not detectable initially had the poorest outcome (*n *= 83; mortality rate 93%).

Trauma patients who received CPR had much more severe injuries, had more frequently head injuries, were more often in a state of shock, and underwent considerably more preclinical interventions such as catecholamine administration, endotracheal intubation, chest drainage, and volume substitution (Table 2).

Patients who needed in-hospital CPR received much larger amounts of blood transfusions than those with pre-hospital CPR, and they also required emergency surgical interventions much more often.

Patients who were discharged alive after pre-hospital CPR had various conditions: only 24% of survivors were able to be discharged at home, while the remaining patients were transferred to a secondary hospital or to rehabilitation clinics (Table 2). Persistent vegetative state (PVS) was observed in 4.9% of patients.

**Figure 3:**
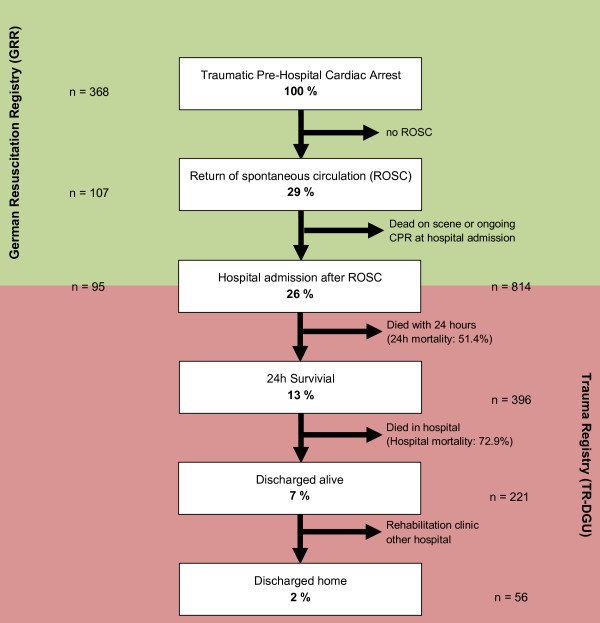
**Summary of the results from the German Resuscitation Registry (GRR) and the Trauma Registry of the German Society for Trauma Surgery (TR-DGU) for patients with traumatic cardiac arrest in whom CPR was started (defined as 100%)**. CPR, cardiopulmonary resuscitation; ROSC, return of spontaneous circulation.

Figure 3 summarizes the results from both registries, with primary outcome calculated for an arbitrary group of trauma patients with cardiac arrest in whom CPR was initiated (defined as 100%). ROSC was achieved in 29%. Excluding patients who subsequently died pre-hospital or who had ongoing CPR on admission (3%), 26% of patients were admitted to hospital with spontaneous circulation. About half of these patients died within 24 hours, resulting in 13% survivors beyond 24 hours. Only 7% of the patients survived until hospital discharge.

The rate of cardiac arrest due to traumatic causes was 3% within the GRR comparable with rates reported internationally [12]. A direct comparison to the Trauma Registry is reasonably difficult due to differing inclusion and exclusion criteria in these two registries. However, data derived from these two registries may reflect the extent to which a good neurological outcome may be possible even in this group of cardiac arrest patients with a very poor prognosis.

Analysis from the GRR database revealed that preclinical CPR attempts resulted in a rate of 25% trauma patients with spontaneous circulation at hospital admission. However, follow up within the TR-DGU showed that hospital discharge rate is only 7%, with 1 of 50 patients being able to be discharged with a good neurological outcome. These results are much worse compared with patients with cardiac etiology that have been reported previously [13,14]. In a review covering a 20-year period in Sweden, Engdahl et al. reported similar low rates for ROSC and hospital admission in trauma patients with cardiac arrest [15]. Despite the poor outcome rates in our study, however, we strongly suggest that pre-hospital CPR attempts should not be withheld in patients with cardiac arrest due to trauma [16,17], as one patient out of 50 young adult patients can be discharged with good neurological outcome.

In addition, David et al. demonstrated in a study conducted in France, that aggressive and intensive therapy in these patients may be worthwhile [18]. The authors reported a ROSC rate of 34% and a hospital admission rate of 30%.

These results also support our thesis that active CPR attempts after pre-hospital cardiac arrest may be as important in trauma patients as in medical patients, in particular if senior physicians are involved in patient's care [18]. Pickens et al. showed a discharge rate of 7.6% in patients with trauma-related cardiac arrest, and further criticized recommendations that suggest not to perform CPR attempts in trauma patients [19]. These results deserve even more attention because the Seattle emergency service was a paramedic system that did not include emergency physicians.

The EMS described in the present study includes pre-hospital emergency physicians working together with the emergency service staff. This system further allows invasive interventions at the pre-clinical scene or during transport, such as chest drain insertion and endotracheal intubation, which are suggested to have positive effects on initial survival in trauma patients [20]. The present study identified differences in volume substitution therapy between trauma and cardiac patients, despite the fact that there were no significant differences either regarding the time at which care was provided at the scene, the arrival time of the emergency services, or bystander resuscitation attempts. Nevertheless, pre- and inhospital treatment should be considered as fast as possible. As some of the early deaths following injury are due to truncal hemorrhage, fast pre-hospital and inhospital treatment should also be recommended. There may be differences in blunt or penetration trauma. Nevertheless, reversible causes of cardiac arrest should be solved as soon as possible, following the international guidelines for resuscitation.

In the study conducted by Engdahl et al. in Göteborg, significant differences were also observed with respect to patient age, location of the event, and initial ECG [15]. Trauma-related cardiac arrest happens more often in much younger patients. In the present study, trauma patients were 15 years younger compared with patients with cardiac causes, and the proportion of patients aged over 60 years was 45% compared with 72% younger than 60 years. This difference in age was also found by David et al. [18]. The location of cardiac arrest also differed. More than 50% of all trauma-related cardiac arrest occurred in public places, suggesting that these patients were physically active until the onset of the cardiac arrest.

### Limitations

On the basis of the requirements and definitions given in the Utstein-type protocol, most publications on CPR exclude cardiac arrest patients with non-cardiac causes. This substantially reduces the number for comparable publications.

Utstein recommended different end-points for describing the success of resuscitation after cardiac arrest. As the GRR does not focus on outcome following hospital admission, we stopped analysis at the time of hospital admission in the present study, but used data from the TR-DGU database for in-hospital follow up. The addition of further demographic variables, patient-related factors such as pre-existing diseases, and laboratory variables may lead to further improvements in the outcome. However, these variables were not recorded and therefore could not be included in the analysis.

In Germany, the EMS are staffed by physicians from several disciplines (mainly anesthesia, surgery, and internal medicine) who have additional training in emergency medicine. This structure allows more pre-clinical invasive technical interventions and the administration of drugs. It further allows the option of stopping any resuscitation attempts at the scene by declaring the cardiac arrest victim as dead. In this respect, the German system, however, does not allow comparisons with the corresponding results of paramedic-based EMS in terms of therapy or outcome.

Finally, documentation of patients within the TR-DGU was stopped when the patient was discharged from the acute-care hospital. It would be extremely valuable to have further follow-up data of these patients, but this is not allowed currently due to the anonymity of data collection.

## Conclusions

In contrast to some trauma management programs [4] suggesting that patients with cardiac arrest caused by severe trauma may not have any chance of survival, our present study encourages CPR attempts in cardiac arrest patients following trauma. In the individual situation, the decision on whether to start resuscitation or not should be made liberally using comparable criteria as for cardiac patients. This is more important, because trauma patients are usually much younger than cardiac patients. However, this does not mean that patients who have injuries that are obviously not compatible with any chance of survival should undergo any resuscitation attempts. In this respect, trauma management programs that contains a more liberal algorithm supporting resuscitation attempts [21] should be encouraged.

## Key messages

• Cardiac arrest caused by severe trauma is a rare situation

• Long-term survival with good neurological recovery is reported in up to 2% of patients

• Starting CPR may be worthwhile in patients with cardiac arrest following trauma

• Trauma management programs that undervalue CPR after trauma should be discussed critically

## Abbreviations

ATH: admission to hospital; CPR: cardiopulmonary resuscitation; DGAI: *Deutsche Gesellschaft für Anästhesiologie und Intensivmedizin*, (German Society of Anaesthesiology and Intensive Care Medicine); DGU: *Deutsche Gesellschaft für Unfallchirurgie *(German Society for Trauma Surgery); ECG: electrocardiography; EMS: emergency medical services; GRR: German Resuscitation Registry; ISS: Injury Severity Score; PVS: persistent vegetative state; ROSC: return of spontaneous circulation; SD: standard deviation; TR-DGU: Trauma Registry of the German Society for Trauma Surgery.

## Competing interests

JTG, JW and MF are members of the steering committee of the German Resuscitation Registry. SS is an associated medical student working in the German Resuscitation Registry. TP and RL are members of the steering committee of the Trauma Register-DGU. All authors declare that there are no competing interests.

## Authors' contributions

JTG, PM and JW made substantial contributions to conception and design, and drafted the manuscript. SS and RL provided statistical support. MF conceived of the study, and participated in its design and coordination and helped to draft the manuscript. BB was involved in the internal reviewing process. TP and AW contributed data to the TR-DGU and helped to revise the manuscript. All authors read and approved the manuscript.
